# Private health insurance in Germany and Chile: two stories of co-existence, segmentation and conflict

**DOI:** 10.1186/s12939-018-0831-z

**Published:** 2018-08-03

**Authors:** Andres Roman-Urrestarazu, Justin C. Yang, Stefanie Ettelt, Inna Thalmann, Valeska Seguel Ravest, Carol Brayne

**Affiliations:** 1 Institute of Public Health, University of Cambridge, Forvie Site, Robinson Way, CB20SR, Cambridge, UK; 2 Department of Health Services Research and Policy, London School of Hygiene and Tropical Medicine, 15-17 Tavistock Place, London, WC1H 9SH UK; 3 LSE Health, London School of Economics and Political Science, Houghton Street, WC2A 2AE, London, UK

## Abstract

**Background:**

In Germany and Chile, substitutive private health insurance has been shaped by its co-existence with statutory social health insurance. Despite differences in the way choice is available to users in the health insurance regimes of Chile and Germany, the way in which each country has managed choice between private health insurance and statutory social health insurance provides a unique opportunity to comparatively assess the consequences of such an arrangement that has been previously underexamined.

**Methods:**

We conducted a Most Similar Systems Design comparative policy analysis of the co-occurring private health insurance and statutory social health insurance systems in Germany and Chile. We describe and review the origins and development of the German and Chilean health care insurance systems with an emphasis on the substitutive co-existence between private health insurance and statutory social health insurance. We provide a critique of the market performance of the private health insurance regime in each country followed by a comparative assessment of the impact of private health insurance on financial protection, equity, and risk segmentation.

**Results:**

Segmentation of insurance markets in both Germany and Chile has had significant consequences for equity, fairness, and financial protection. Due to market failures in health insurance and differences in the regulatory frameworks governing public and private insurers, the choice of public or private coverage has produced strong incentives for private insurers to select for risks, compromising equity in health care funding, heightening the financial risk borne by public insurers and lowering incentives for private insurers to operate efficiently.

**Conclusions:**

The degree of conflict arising from the substitutive parallel private health insurance system and the statutory social health insurance system varies between Germany and Chile, though policy goals remain similar. Recent reforms in both countries have attempted to improve the financial protection of the privately insured through regulation; nevertheless, concerns about risk segmentation remain largely unresolved.

**Electronic supplementary material:**

The online version of this article (10.1186/s12939-018-0831-z) contains supplementary material, which is available to authorized users.

## Background

In Germany and Chile, substitutive private health insurance coexists with social health insurance schemes which cover the majority of their respective populations [1–4]. Although only a minority of the population in each country is covered by private health insurance, the choice between private and social insurance plays an important role in both countries’ health systems and crucially shapes the boundaries between the private and public health systems, and the regulation, financing and provision of each [5, 6]. One of the most discussed results of choice between private and social health insurance is risk segmentation [7]. In both countries, it has been argued that this phenomenon is a direct consequence of choice. The funds from those who are able to afford and choose private health insurance are pooled separately from mandatory social health insurance wage deductions, creating strong incentives for private insurers to select for those who can afford private health insurance [4, 8]. In Germany, the choice of opting out of the statutory system is restricted to those with an income above a certain threshold. Once an individual has chosen private insurance, the option of returning to the social health insurance regime is limited. In Chile, the choice of private insurance is dependent only on ability to pay because private plans are considerably more expensive than contributions to the public system and there are no limitations on who may or may not purchase private health insurance as in the German case [9]. However, the privately insured in Chile may re-join the statutory system at any time, an option which is prohibited in Germany to reduce the potential for further risk segmentation.

Taking into account the different dimensions of regulation, financing and provision [10], choice and the free flow of users between insurance regimes [7], the main objective of this comparative policy analysis is to analyze how the designs of the German and Chilean substitutive private health insurance regimes have led to risk segmentation and created financial barriers to health coverage [5, 7, 10]. We decided to base our analyses on Germany and Chile based on a Most Similar Systems Design (MSSD) [11] because: a) they present crucial similarities in their respective health system designs, such as the substitutive nature of both private insurance regimes, b) the public-private insurance mix, although different, has the potential to inform discussion around policy reform in both countries and their World Health Organisation (WHO) sub-regions, c) the choice between public and private insurance has been available for significant periods (from 1970 to the present day in Germany, and since 1981 in Chile), with Germany being the only country in Europe still allowing substitutive choice [4, 7]. Moreover, in the context of universal health coverage, various countries have considered emulating the German model such as Italy, Portugal and the United Kingdom in the 1990s, and Croatia, Portugal, Russia, Slovakia and Slovenia in the 2000’s [7, 12–14]. Debates about the choice between public or private insurance in these countries have been framed in terms of offering some, or all, of the population the possibility of opting out of the public insurance scheme [7].

This analysis deals with current policy debates which may overstate the potential benefits of choice in insurance markets [7]. Another benefit of comparing both countries is the opportunity to assess the ways in which differing policies on the flow of users between private and publicly insurers in Chile and Germany may elucidate the ways in which these policies have played crucial functions in how risk segmentation has been tackled. We expand on this, arguing that in Germany and Chile the choice of social and private health insurance has jeopardized equity and fairness, with far-reaching consequences for their health systems. We define equity in health care as equality of utilization, distribution according to need, equality of access, and equality of health [15–17]. We are interested in discussing the effect that risk segmentation has on equity, and whether the negative effects on equity might be expressed more strongly in a country that allows a free flow of users, such as Chile. We focused on equity and efficiency as primary outcomes because these particular analytical concepts are key to WHO’s health policy goals for health insurance [18].

## Methods

Our study of health care insurance policy in Germany and Chile adopts a comparative case study approach based on two countries using the MSSD [11]. Under the MSSD framework, comparative policy analysis proceeds with the selection of cases which share key salient features (e.g. policy design, sociodemographic characteristics) and an analysis of divergent policy outcomes. Given our interest in the interface between statutory health insurance and private health insurance, specifically systems in which beneficiaries may transition between public and private coverage, we selected Germany and Chile for our analysis. Germany and Chile share similarities in their respective organisation of choice between public or private health insurance and, moreover, the way in which they have organised the boundaries between them may inform future policy developments and health system reform in both Europe and South America [11].

We begin by tracing the origins and development of the modern health insurance regimes in each country respectively before engaging in a comparative policy analysis of each country’s approach to regulating health insurance and its divergent consequences on health equity and efficiency. We adopt a mixed methods approach, integrating both qualitative and quantitative data from the literature where available; nevertheless, we acknowledge the difficulties of direct comparisons between these two disparate countries. As such, we conducted a focused literature and policy documents search and used a thematic approach to analysis, in line with the research question, highlighting empirical findings from the literature to support comparative statements between Germany and Chile where possible. Documents were collected thematically in English, German and Spanish by searching for “private health insurance” and “social health insurance” adding then “Germany” and “Chile”. In the case of Chile we searched directly for “FONASA” and “ISAPRE” and in the case of Germany for “GKV” “PKV” from PubMed, Google Scholar. We also searched from the OECD document repository together with documents in the public domain such as policy releases and comments. Searches were conducted between March 2015 to November 2017 and reviewed for relevance and currency. Please see PRISMA chart for details of search results (Additional file 1).

### Framework for analysis

In order to outline the role that private insurance plays in Germany and Chile, we will start by defining it according to the taxonomy of private insurance proposed by the Organisation for Economic Co-operation and Development (OECD), which states that primary private health insurance is private insurance which represents the only available access to basic health cover because either individuals choose not or are not entitled to have public health insurance [19]. Using the same OECD taxonomy for classifying health insurance systems, in both countries being examined private health insurance can be classified as primary substitutive, which is to say, private health insurance is substituted for cover which would otherwise be offered through social insurance or a publicly financed insurance scheme [19].

To assess regulation, financing and provision of health care/insurance in Germany and Chile, our comparative analysis will use the framework proposed by Wendt and Rothgang [5, 10]. Wendt and Rothgang’s deductively generated framework classifies health systems according to the three dimensions of regulation, financing, and provision of health care/insurance, yielding ten theoretically plausible types of health care systems where only five system types are observed in practice: the National Health Service, the National Health Insurance, the Social Health Insurance, the Etatist Social Health Insurance, and the Private Health System [5, 10]. One of the reasons for comparing both countries is that they have both provided a choice of healthcare plans for those able to afford them. The dynamic between choice and the different aspects that it entails is important to analyse due to its effects on efficiency and equity. For assessing the interaction between the three dimensions (regulation, financing and provision) and choice, we will use the framework provided by Thomson and Mossialos [7] which analyses the impact of giving consumers choice between public or private health insurance. Specifically, Thomson and Mossialos highlight the ways in which arguments by proponents of consumer choice in health care, who usually argue that consumer choice among health plans promote competition between public and private insurers, are undermined by market failures unique to health insurance markets, primarily involving information asymmetry [7]. Thomson and Mossialos detail an approach where policy examination focuses on whether or not they have engendered risk segmentation and whether or not they have created financial barriers to private coverage [7].

In this paper, we hypothesize that allowing a substitutive choice of insurance is likely to result in two main outcomes: a market segmented by degree of risk and by financial barriers to private coverage for high risks. We advance the argument that private health insurance suffers from serious market failures largely due to asymmetries of information that tend to negatively affect efficiency and equity in health systems [20]. Furthermore, health insurance markets affected by adverse selection tend to be inefficient because they deter people with low health risks from buying comprehensive coverage, without cost sharing and at a fair price [7, 21]. Additionally, the threat of adverse selection creates incentives for private insurers to use risk selection as competitive strategy, by attracting people with low health risk and excluding those with high risk [7]. Our comparative analysis will follow the same definition of equity and efficiency used by Thomson and Mossialos [7], acknowledging the multiple interpretations of equity and efficiency that are associated with diverse cultural and societal values [7, 21]. This work compared choice of public or private health insurance in Germany and The Netherlands, two countries with similar socio-demographic characteristics and income levels while also both being Member States of the European Union. So far the comparison of high middle income countries like Chile with high income countries like Germany has not been performed and might prove crucial to define health reform discussion in the EU and beyond. In our work we will compare two countries with different levels of income that offer substitutive private health insurance in order to assess whether individuals are treated equally based on how public contributions or private premiums are determined by features such as health status, income, age and household size as indicators of horizontal equity [7].

For both Germany and Chile, we analyse the degree of progressivity in financing health as an indicator of vertical equity by assessing whether those who receive higher incomes pay proportionately more than those with lower incomes [7]. For productive efficiency, usually defined as outputs achieved from given inputs, we will discuss how competition is based on risk selection rather than price and/or perceptions or quantitative measures of quality in Germany and Chile [7]. Productive efficiency will also be assessed by examining how private insurers reduce operating costs by lowering their administrative costs, and to what extent insurers try to decrease premium prices by adjusting provider fees and influencing provider behaviour [7, 20, 22]. We will include both empirical and qualitative data in this review in order to compare both countries using the framework proposed by Wendt and Rothgang Our analysis takes a long-term perspective, beginning with the creation of PHI in the 1880’s for both countries to then be organised and follow the most important policy milestones of health insurance reform in both countries in the last 50 years.

## Results

### Private health Insurance in Germany and Chile

From the searches of policy documents and databases we found Records identified through database searching (PubMed & Google Scholar) we were able to retrieve 24 publications and 55 additional records identified through other government or policy sources such as OECD. We then screened 79 records removing 4 duplicates removed and assessing 75 full-text publications for eligibility. Three publications were excluded, because of falling outside the scope of our work. 72 studies were then finally included in our qualitative synthesis.

In Germany and Chile private health insurance has historically developed alongside statutory health insurance. In Germany, statutory health insurance was made mandatory for industrial workers in 1884, constituting the first national social insurance scheme of its kind [2]. All other population groups were formally excluded from statutory health insurance at the time and could only obtain health care coverage privately, if at all. Statutory insurance has expanded over time and since 1989, all workers with earnings above a certain threshold were given a choice between statutory health insurance (Gesetzliche Krankenversicherung - GKV) and private coverage (Private Krankenversicherungen - PKV) except for civil servants who have always had private coverage in addition to support from the state.

Under current legislation, private health insurance is required to comprehensively cover both ambulatory and hospital care, although insurance plans vary with regard to other forms of health care (e.g. dentistry, eye care, alternative medicine). Since 1994, individuals over the age of 65 years (55 years since 2000) are legally prohibited from returning to the GKV once they have decided to opt for PKV, even if their earnings drop below the income threshold. This measure was introduced to protect sickness funds from further risk segmentation resulting from younger people opting for private coverage and re-joining the GKV once they are older and find their private premiums more than contributions to GKV.

Health insurance in Chile came to existence in the 1880s inspired by the same Bismarckian principles of social protection stemming from Germany [23]. In 1952 Chile created the Chilean National Health Service (Servicio Nacional de Salud - SNS) after the British National Health Service with a complementary insurance regime for private users and white-collar workers. This system was in place until 1980 when after a right wing military coup that toppled the Socialist President Salvador Allende, a set of health reforms imposed by the military junta led by General Augusto Pinochet created the legal and institutional framework for substitutive private health insurance. It stipulated that workers in the formal economy could choose to pay a mandatory 4% of their taxable wage income into a public or private insurance fund [24]. Individuals were able to choose between joining the centralised statutory health insurance scheme called National Health Fund (Fondo Nacional de Salud - FONASA) or taking out private health insurance premiums from a variety of different for profit companies collectively named Private Health Institutes (Institutos de Salud Previsional - ISAPRE) [25]. This policy enacted significant changes in the financing of health care in Chile and led to risk segmentation with important consequences for the overall equity of the health system [26, 27]. For example, by 1988 the ISAPRE system contributions had increased from 4 to 7%, covering only 11% of the population; yet they collected more than 50% of the mandatory health care contributions, accounting for 38% of the total health care expenditure [25].

After 17 years of neglect caused by the structural adjustment programme of the military dictatorship and with the country’s return to democracy in 1990, health reform became a top priority for the newly elected democratic government [24]. President Patricio Aylwin enacted an ambitious infrastructure programme while also improving the work conditions of the health work force during the 1990s [24]. From 2003 to 2004, President Ricardo Lagos designed a reform package addressing the problems arising from the parallel existence of private and statutory health insurance [28]. The reforms tackled what was seen as the serious problem of risk segmentation of the insurance market and attempted to increase financial protection across the private and public insurance sub-systems [1].

The most important bill of the reforms was the 2005 ‘Long Law’ of ISAPRE. This bill defined a state guaranteed set of fully covered medical conditions based on disability-adjusted life years (DALY) for both the public and private health insurance regimes [1]. This was termed the System of Explicit Health Guarantees (Garantias Explicitas en Salud - GES). It ensured that ISAPRE plans would provide a basic price for each of the existing plans (which currently number around 64,000), differing in its total price depending on the risk profile of the user while maintaining any price raise within a 30% threshold of the mean price for a private health plan. It determined a set of clinical guidelines and pathways, introduced preset periods for diagnosis, treatment, or follow-up with a maximum of out-of-pocket expense cap and waiting times, after which private providers for the services could be used [29]. The number of conditions covered was expanded from 2 in 2004 to 80 in 2015 and eventually has included all major disease areas which carried the highest burden of disease and disability in Chile [26, 27]. In 2005, further legislation introduced rules for annual premium changes that were to be calculated from a table of pre-defined risk factors, which were already in use by private insurers [30]. However, the table of risk factors was declared unconstitutional by the Constitutional Tribunal in 2010, who deemed the table to be discriminating according to gender and age and creating a crucial legal challenge to ISAPRE [31]. So far ISAPRE are still able to use the table of risk factors to set annual premium charges for people changing insurance carriers and new affiliates. The current government of President Sebastian Piñera has avoided regulating this issue and a final policy resolution in this matter is still pending.

### Regulation

Health insurance in Germany is heavily regulated through legislation. Social Code Book V (Sozialgesetzbuch - SGB V) regulates all aspect of statutory health insurance, including criteria for eligibility. While SGB V does not regulate private health insurance directly (perhaps apart from the ‘basic tariff’ and the price setting for Diagnosis Related Group - DRG in the hospital sector), changes in legislation aimed at reforming the GKV often affect private health insurance. In addition, private health insurance is regulated through several laws and ordinances that apply to the insurance market in general (e.g. the insurance contract law) or to private health insurance specifically (e.g. provisions for savings). Financial oversight of the private health insurance market is exercised by the Federal Supervisory Office for Financial Services (Bundesanstalt für Finanzdienstleistungsaufsicht BaFin), an agency of the Ministry of Finance. Developments in the private health insurance sector are also closely observed by the Ministry of Health, although the Ministry has little direct control over the market. Private health insurance also qualifies for tax subsidies, as does statutory health insurance and other forms of insurance. Since January 2010, a special tax subsidy applies exclusively to health insurance, covering both private health insurance premiums (for health services equivalent to the regular GKV benefits package) and GKV contributions, again not privileging private insurance [32].

Since 2005, changes in the price of premiums in Chile are regulated by the newly created Health Superintendence (Superintendencia de Salud), which became the government’s main regulatory body for ISAPRE and FONASA. This body sets a basic premium that centrally determines the scope for annual increases in premiums. ISAPRE are bound by this base premium and have to set premiums within a 30% ‘premium band’, limiting their ability to differentiate premiums according to different types of plans or customers [33]. In addition, the Health Superintendence laid down rules in regards to the risk factors used to calculate premiums, legislation which was controversial and particularly opposed by ISAPRE but was deemed discriminatory in 2010 [30, 31]. A risk equalization fund redistributes resources between ISAPRE adjusting for the financial risks associated with factors such as gender, age, diagnostic categories, and morbidity, as a mechanism to fund the community-rated explicit guarantees [34]. This mechanism aims to compensate private insurers for the income lost because of the new regulatory framework and the costs associated with the introduction of explicit guarantees. It also aims to provide an incentive for insurers to reduce risk selection. So far, the impact on the ISAPRE of this risk compensation fund has been negligible, considering that GES benefits for ISAPRE users were care packages that did not allow choice of providers which many users considered an important factor in their decision to choose private health insurance. This, among other issues, was exploited by ISAPRE public relations campaigns aimed at reducing the impact that GES might have on the private subsystem. All these issues make a strong case for the fundamental difference between countries being related to the type of regulation and the levels at which it operates. Considering Germany is a Federal country with a much larger population and a sophisticated health policy agenda, Chile faces far stronger opposition to thorough and extensive reform of the current system.

### Financing

Although similar in their public-private health insurance configurations, there are important differences between Chile and Germany. These include respective gross domestic product (GDP) per capita, health expenditure as proportion of GDP, the size of the private insurance market and the amount of private spending [35, 36]. For example, total spending on health care in Germany was 11% of GDP in 2013, 76.3% of which was public funding [35]. About 87.5% of the population (2011) are members of a GKV fund [37]. In addition, about 11.7% are covered by substitutive private health insurance [38]. The remaining recipients of subsidies (Beihilfe) are civil servants, members of the armed forces, and recipients of social benefits or a veteran pension. Self-employed individuals are not required to join a sickness fund and usually take out private health insurance, as they would otherwise have to pay both the employer’s and employee’s share, which makes GKV membership unattractive to them. Civil servants only have to cover the remaining percentage of health care costs, for which they can take out private insurance coverage.

There are two types of substitutive, private health insurance plans: Full Insurance (Vollversicherung), which provides full coverage of the costs of health care equivalent (or higher) to those covered under statutory health insurance, and additive premiums (Zusatzversicherung), which typically provides complementary or supplementary private health insurance coverage for the cost of health services excluded from statutory health insurance or co-payments. In 2011, about 22.5 million people took out complementary/supplementary private health insurance, as compared to 13.8 million in 2000 [39]. Private health insurance products are currently offered by 41 insurance companies, with 12 insurance companies having a joint market share of 76.89% [38, 40]. 24 of these are publicly listed corporations, usually with a wider insurance portfolio, 17 are Mutuals specialised in health care and 11 are subsidiaries of mutual organisations [38, 40].

Premiums in the public system are currently set at a 14.6% split between employers and employees [41]. The employer’s share has been fixed at 7.3%, which means that all future increases will be borne by the employee in order to limit the burden on labour costs for employers [41]. Sickness funds are not allowed to discriminate among members, which is to say, all members of a fund must be charged the same rate. For certain groups, such as the long-term unemployed on benefits, GKV contributions are paid by public authorities. Since 2009, GKV contributions are centrally pooled, in a virtual ‘health fund’, and distributed between sickness funds adjusted for age, sex and morbidity. Health insurance became mandatory in January 2009, although coverage was almost universal before then with only about 0.2% of people being uninsured in 2007 [37].

In Chile, about 76.3% of the population was covered by FONASA, while 18.2% voluntarily took out ISAPRE [42, 43]. The remaining 2.95% of the population had institutional coverage, such as the armed forces [42, 43].In the case of Chile, total health care expenditure in 2013 was significantly lower than in Germany, and accounted for 7.3% of GDP of which 46.6% was publicly funded. This was in stark contrast to Germany’s large share of public expenditure, which may be expected with a private insurance market half of Chile’s size [35]. In 2015, there were 13 ISAPRE companies, with the three largest having a market share of almost 60% [44]. There is also a growing market for complementary health insurance, with 12.3% of the population having purchased some sort of complementary insurance plan by 2010 [44]. However, complementary insurance is offered almost exclusively through group contracts, which constitute around 87% of the total market [45].

In Chile, out-of-pocket (OOP) expenditure has created serious barriers to health care access and use [9]. Out-of-pocket payments as a percentage of total health expenditure remain remarkably high in Chile (32.4%) as compared to Germany (12.9%) and the OECD average (19%), and are one of the main barriers of access to health and a serious determinant of catastrophic expenditure [46, 47]. Households that face difficulties paying medical bills may delay or even forgo needed health care [48]. This is evidenced by the fact that ISAPRE users have larger OOP payments than FONASA users (respectively 6.1 and 3.8% of their income in 2013), not only in absolute terms, but also in terms of the share of their income devoted to direct payments [9, 49].

### Eligibility, benefits and premium conditions

Eligibility for substitutive private insurance in Germany is limited to those not mandatorily insured under GKV. By 2018 GKV membership is mandatory for employees with earnings below €59,400 a year, some self-employed professions such as farmers, artists and journalists, students, those receiving unemployment benefits, people with a disability (if they work in a recognised institution) and retired people who were a member of a sickness fund prior to retirement. Employees who have earned above the threshold for one year and their dependants (about 20% of the population) can opt for substitutive private coverage, which exempts them from contributing to GKV.

In Germany, private health insurance premiums are based on an assessment of an individual’s risk profile at the time of purchase. Variables considered include age, sex and the medical history of the applicant. For employees, the cost of the premium is typically shared with the employer. The employer’s share includes premiums for the insured and his/her dependants. It is set at 50% of the rate that employers and employees would have to contribute if the employee were GKV-insured and is capped at 50% of the actual insurance premium [50]. Dependants are not automatically covered and must pay separate premiums. Cover is for life and operates on a funded basis. Since 2001, insurers have been required to build up ‘ageing reserves’ to cover age-related increases in costs (and slow the increase of premiums) later in life – these reserves are built by charging all clients between the age of 21 and 60 an additional 10% of all premium payments made.

German insurers may reject applications and exclude pre-existing conditions from cover or charge a higher premium to cover these conditions. However, since 2009 they are required to accept any applicant (open enrolment) who is eligible for the basic tariff and cannot exclude cover of pre-existing conditions for this tariff. Individuals who have opted for private insurance after January 1, 2009, including people over the age of 55 years, and those receiving benefits or a pension are eligible. The basic tariff covers services provided by GKV at a capped premium (€656 per month in January 2016). If an individual demonstrates an inability to afford the full premium for the basic tariff, the premium will be reduced by 50% and the remainder will be subsidised by the state. If this remains unaffordable, individuals receive a state subsidy under the social benefits scheme. Substitutive private health insurance typically covers the same comprehensive range of benefits as the GKV. Since January 2009, private health insurance plans must cover ambulatory outpatient and short-term inpatient services. Insurers typically impose a waiting period of three months before benefits apply, but this may be waived if a new customer was previously covered by the GKV [51]. Benefits are mainly provided in cash, that is to say, the individual pays for treatment first and is subsequently reimbursed by the insurer. Substitutive contracts may involve cost sharing. For example, co-insurance is common in dental care, where patients pay a proportion of the total costs. Private health insurance plans can also include deductibles, in which the deductible amount has been capped at €5000 per year [50].

Private health insurance in Chile is available to those who can afford private premiums. Historically, it has been based on wage payments that in 2018 should not exceed a predefined maximum per calendar month of €210, which is the same cap set on premiums for users in FONASA [52]. However, if the mandatory wage deduction does not cover the premium price, the remainder has to be paid on top of the 7% contribution. Companies offering private plans are also allowed to negotiate wage-based contributions with customers beyond the statutory ceiling if they wish to offer supplementary services or a larger choice of providers [53]. Contracts with ISAPRE are usually for one year and extensions are subject to review based on tables of individual risk factors, while ISAPRE users are also allowed to re-join FONASA unconditionally [24]. Private premiums are high relative to contributions to FONASA’s four members categories, which are paid mandatorily as 7% of income. FONASA categories A and B include people no income or with a monthly income below €345 for affiliation and are exempt from making contributions to FONASA. FONASA category C includes people with a monthly income between €345 and €505 who pay a 10% copayment fee. People with an income higher than €505 per month are included in FONASA category D which has a 20% copayment fee. By law, ISAPRE is required to cover at least the same services covered in FONASA’s two lower income plans [25, 30]. A contribution of 7% for the chilean average monthly income in 2016 of €694 to FONASA D would thus equal to around €49, regardless of the risk and sex of the user [36]. The cheapest ISAPRE premium is are in the range of €55–60 monthly and includes similar or lower coverage than FONASA D for a 30 year old low-risk single male individual earning the the chilean average monthly income [54, 55]. It is also crucial to define the progressivity of health financing in Chile considering that wage deducted contributions have historically covered less than 60% of FONASA’s budget. In 2013, this resulted in only 42.5% of FONASA members making wage deducted contributions to the system, while the remaining funds were supplemented by direct government transfers [56]. Consequently, without a progressive tax system that would consider the makeup of corporate and income contributions, the proportion of sales taxes in the overall Chilean budget, and the degree of tax evasion, any direct State transfer to FONASA might be regressive in nature.

### Provision of health services

Health care delivery is organised in Germany through a range of health service providers, including public, private not for profit and for profit organisations. Indeed, pluralism of provider ownership is a statutory principle of the German health care system and patients have a choice of provider, regardless of their insurance status. Hence, both GKV members and the privately insured can access (almost) any provider, including public and private (for profit and not for profit) providers. Private insurers – like sickness funds – are largely bound by collective agreements as to provider payments formed by the associations of sickness funds and provider associations (i.e. German Hospital Association and Associations of GKV Physicians). In addition, they can form agreements with providers that only treat privately insured patients. Vertical integration with providers is rare and not permitted in some cases. For example, insurers are not allowed to own polyclinics.

Private health insurers generally must match market prices. In the hospital sector, prices per service are reimbursed based on DRG and prices are identical for statutory and private health insurance. In ambulatory care, prices are based on a list of ‘basic prices’ issued by the Federal Ministry of Health. However, physicians can charge higher fees by multiplying the ‘basic price’ with a factor set to reflect the level of complexity and time for treatment (e.g. a factor of up to 3.5 for personal services rendered by a physician and 1.3 for laboratory services). Physicians are also allowed to bill in excess of these prices, although this requires approval of the insurer before the service is provided [57]. Unlike sickness funds, insurance companies only form direct contractual relationships with clients, not with providers. Consequently, private insurers have little leverage over providers, many of whom can charge higher fees for privately-insured patients than for members of sickness funds. For instance, Waldendzik et al. [58] have demonstrated that prices for physicians’ services are more than twice as high for the privately insured than for those covered by statutory health insurance.

In Chile, ISAPRE and FONASA are separate providers. However, FONASA has historically bought services from private providers with pay as you go vouchers and more recently, as part of the explicit guarantee regime, which established that if guarantees were not met in time by public providers, users could choose to use private providers. FONASA’s pay as you go vouchers are part of the Free Choice Modality (Modalidad de Libre Eleccion - MLE) that allows those FONASA users in the upper segments (C and D) to use private providers while incurring an increased percentage of copayment. This is why one can observe a crossover between public and private providers, where ISAPRE users that are underinsured tend to use public providers for catastrophic events and wealthier FONASA users tend to use the MLE and pay as you go vouchers to increase their choice and access to outpatient services. ISAPRE generally pay private providers on a fee-for-service basis, while FONASA tends to pay public providers in the state run subsystem based on a list of hospital and physician fees, which are centrally defined by FONASA and the Ministry of Health. Conversely, most ISAPRE allow free choice and tend to use market pricing for paying their providers while, in some cases, using lists of preferred providers and negotiating prices for them in bulk. ISAPRE have increasingly merged with providers, that is to say, integrating them into health care holdings and encouraging their use by a series of financial hedges. Providers in this financing framework have a strong incentive to over-provide services to certain private patient groups. For instance in 2017 the rate for a caesarean section were persistently higher for privately insured women (69%) than for those who delivered in public hospitals (40.9%) with a global caesarean rate of 46% according to OECD [59, 60]. It is important to note also the high prevalence of caesarean sections in public providers. Escalating health care costs have been the consequence of using fee-for-service as method of payment under private insurance. Simultaneously there has been increasing public pressure on ISAPRE for what people regard as their excessive profits in the private subsystem [61]. This is seen as one of the reasons why ISAPRE have increasingly merged with private providers as a way of both transferring profits to them and controlling the cost of health services, while maintaining the profit levels of integrated health care clusters in an increasingly competitive market [61]. The main consequence is the emergence of managed care clusters, similar to the vertical integration of providers such as Health Management Organisation (HMO) in the United States [62]. Health care holdings linked to ISAPRE owners control about 42% of the private provider market, even though vertical integration is explicitly forbidden by law since 2005 [61, 62]. This prohibition stems from the fact that in cases of vertical integration the insurer can manipulate demand for health services and steer patients to the most profitable integrated provider. So far, vertical integration has not been challenged in Chile, despite the abundant international evidence of how service integration and fee-for-service incentivise overprovision and stimulate cost inflation.

### Recent policy developments

While substitutive private health insurance has been a source of controversy in Germany for decades, the dual insurance system – often dubbed two-class medicine (Zweiklassenmedizin) by its critics – is fiercely defended by the medical profession. As the political costs of change are high, changes are largely introduced at the margins of the system. For example, the 2007 reform introduced optional tariffs (Wahltarife) within the GKV, which allow sickness funds to offer a more diversified range of insurance plans, such as those with deductibles, which previously had only been offered to those with private coverage. These new tariffs have at least in part aimed to attract or retain those who are able to choose between GKV and private coverage.

Following the coming into force of the Act to Promote Competition within the GKV (GKV-Wettbewerbsstärkungsgesetz) in 2009, health insurance, public or private, became mandatory for all residents. Concurrently, private insurers were required to offer a ‘basic tariff’ (see eligibility, benefits and premium conditions). Since 2009, customers have been allowed to take the portion of the ageing reserve attributable to the basic tariff with them if they change insurance company (or the entire reserve if they change plan within the same company). The aim was to reduce the barriers for customers to switch between private insurers and to promote competition among companies. It is important to note that competition is restricted to those who can voluntarily join statutory insurance. This is a crucial issue in the German system, particularly considering that it is important to differentiate competition between the two insurance systems from that of competing for privately insured persons alone.

Since 2013, the abolition of out-of-pocket practice fees for the statutorily insured came into force with the aim of restoring equity. Beginning in 2004, the €10 fee that was paid quarterly by GKV for those insured above the age of 18 was aimed at reducing doctor visits. However, no decrease in doctor visits was observed and one unintended consequence of the policy was that it ended up targeting low-income individuals, leading to a decrease in equity. GKV reforms have also focused on regional equity, i.e. introducing financial incentives to encourage the settlement of doctors in underserved and structurally weak regions (Act to Strengthen Care Provision in the Statutory Health Insurance system 2012 and 2015) [63].

Reforms in 2015 focused on the development of the finance structure and the quality of statutory health insurance. Wage-based contributions were lowered from 15.5 to 14.6% in 2015, while allowing sickness funds to charge additional contributions (income-related and without upper limit). By 2018 these range from 0 to 1.7%. In addition, insured members can switch to competing sickness funds more easily if a sickness fund decides to increase additional contributions above the national average, which is to be determined each November (1,0% in 2018) [63]. However, whether this policy will stimulate competition and efficiency in the statutory health insurance market is debateable, given that additional contribution rates are expected to rise in future.

In 2014, a new round of health reforms tried to tackle the entrenched inequity of the system in Chile, which was caused by illnesses and disorders falling outside the explicit guarantee regime and the existence of high levels of co-payments in both parts of the system [34]. This led to the creation of the preferred provider network of the Additional Coverage of Catastrophic Events (Cobertura Adicional de Enfermedades Catastroficas – CAEC) in 2013 for ISAPRE users aimed at dimishing the impact of OOP in catastrophic events and also the Financing System for Diagnosis and Treatment of High Cost Programs (“Ricarte Soto” Law), which was established in 2015 to increase financial protection and catastrophic coverage for those illnesses not included in the explicit guarantee regime for both ISAPRE and FONASA users. The Financing System for Diagnosis and Treatment of High Cost Programs is funded entirely through state contributions and does not require the payment of an additional premium. FONASA currently administers this fund, and all beneficiaries of the Chilean health care system are entitled to use it. This can be interpreted as a covert subsidy to ISAPRE since the central government will be financing the fund. With the same goal in mind, in 2011 a new policy abolished the 7% health care contribution for pensioners over the age of 65 in the four poorest quintiles of the population. Again, those primarily affected FONASA users were older people, and higher risks; which in real terms meant higher state transfers to the public insurance scheme. This policy was intended as a direct cash transfer by the state, aiming at effectively increasing the monthly income of pensioners.

In 2015, the newly appointed Advisory Commission of the Presidency was commissioned by President Bachelet with the mandate of dealing with the relationship between the two subsystems [42]. The Commission recognised that private health insurance reform affects the entire system and produced a series of proposals. The commission was formed as a cross party initiative and developed two proposals presented to the President [42]. These were a majority proposal and an alternative minority proposal. The majority proposal recommended the creation of a Universal Health Fund (Fondo Mancomunado de Salud Universal - FMSU) in the form of a single health insurance and with a single payer mechanism alongside supplementary and or complementary private health insurance [42]. The minority proposal suggested a less disruptive option which entailed continuing with the free choice of private health insurance alongside the state sponsored regime. The Universal Health Common Fund would pool all contributions with direct state transfers that would together finance Chile’s health care system and cover all residents.

The Presidential Commission also proposed creating a Cluster of Health Benefits (Conjunto de Beneficios Sociales - CBS); this would include almost all policies and mechanisms of financial protection such as GES, CAEC and the “Ricarte Soto” Law. Similarly, the Presidential Commission also proposed that inter-ISAPRE discrimination based on pre-existing health conditions should be gradually removed from the entire system. The new insurance premium proposed by the Presidential Comission would still correspond to the legal contribution of 7% per household and allow access to a broad range of providers and a community premium for individuals to access other networks. Unlike the current insurance contracts, price re-adjustments would be regulated on the basis of costs and/or evaluation by expert panels and with capped profit levels.

The Presidential Commission also suggested reducing the number of commercialized plans to three, instead of currently 14,000, to increase the transparency of the market [42, 64]. Additionally, supplementary health insurance would only be allowed for CBS users, restricting the private insurance market considerably [64]. To compensate for the increased health risk, the Inter-ISAPRE Risk Compensation Fund would adjust the total of the CBS and consider the total legal quota. The creation of the FMSU would establish a basic foundation for the solidarity between systems, which does not currently exist. At present, surpluses accumulated by an individual through premium payments are returned to the contributor, while the new law proposes to enter the surplus into a pooled fund. Finally, the implementation of the amendments to the law would be voluntary for current contract holders but mandatory for new contracts. The non-legally-binding majority proposal of the Presidential Commision thus integrated some aspects of the German private insurance regime, such as the Universal Health Common Fund. At the same time, it did not achieve a quorum to support the recommendations producing a minority proposal. So far, it has not been clear whether the task of the Commission or its recommendations will be integrated as part of Chile’s health reform and therefore they remain part of a discussion rather than a solid and consistent policy plan with a path to implementation. The conclusions and recommendations of the Presidential Commission have not produced any change in health policy. One could also argue that the conclusions, being non-binding, follow the long precedent of Chilean Commissions, going as far back as the Indigenous Affairs Commission of 2001 (Comision Verdad y Nuevo Trato), that have been historically aimed at deflecting pressure of social reform processes outside of the Executive and Congress [42, 65, 66]. Most of these Commission’s recommendations have never been incorporated into policy processes and hence have been heavily criticized by different stakeholders [65]. Further proof is given by the fact that during President Michelle Bachelet’s first period between 2006 and 2010, six Presidential Commissions were created (Pensions, Infancy Policy, Quality in Education, Transparency and Corruption, Higher Education and Labor and Equity) [65, 66]. After 2014 and again under President Bachelet’s second government, five newly appointed Commissions were formed of which two were related to the same issues raised in the first government (Transparency and Corruption and Pensions) casting serious doubts as to the effectiveness of the recommendations made by these means [65, 66].

## Discussion

While substitutive private health insurance in both Chile and Germany has developed alongside statutory health insurance, private insurers have carved out distinctive niches for themselves and developed different business models. The political context is such that the political costs for reforming (or abolishing) substitutive private health insurance are significant, as private health insurance is supported by health care professionals and affluent beneficiaries unwilling to forsake the advantages they receive from it. The fact that the above are differences in socio-economic context may also be relevant. In Chile, a middle-income country, public services still face substantial resource constraint, hence the need to ration care using waiting lists. In Germany, high spending on health care (public and private) as well as high consumer expectations have created a climate where increases in the costs of care are a constant concern in the face of an ageing population, even though pressure to reform is (mostly) on the statutory public system.

One crucial common characteristic in Germany and Chile has been the creation of a boundary between the statutory and private insurance regime that enables people to choose between them. Both countries vary in their approach to regulating this boundary. In Germany, the choice between public and private coverage is largely limited to those with earnings above a legally defined income threshold, while in Chile everyone can take out private coverage as long as they can afford it. Additionally, those who have chosen private insurance in Germany face substantial barriers if they wish to return to GKV. In contrast, within Chile everyone has access to publicly funded health care with no restrictions upon those who are privately insured. The choice of countries is predominantly informed by the boundary problem between public and private substitutive health insurance [67].

In the case of Germany, one could argue that open enrolment has been an important tool to increase access in private health insurance and is a missing policy in the case of Chile, with the Presidential Commission highlighting this fact as a desirable policy outcome. However, open enrolment also creates new incentives for adverse selection when favourable risk users initially seek very low coverage and then change to more comprehensive coverage when they happen to develop unfavourable risk factors. It is difficult to reduce or eliminate these incentives for adverse selection.

One way to deal with this would be to limit access to private health insurance to within a limited period, for example after losing or switching coverage from the public scheme to private health insurance. Another way of analysing the differences between statutory and private insurance is in terms of what services are covered and the level of financial protection users have. In Germany, private plans are required to provide coverage for inpatient and outpatient care as covered by GKV at a minimum. In Chile, the 2003 reform has introduced a set of explicit guarantees for the treatment of a set of major conditions that health insurance is required to cover. This new regulation aims to address the previous tendency of Chilean insurers to exclude services and reduce financial protection. However, the policy has not yet been introduced consistently after 11 years of its implementation. This is because its original design failed to address issues like the perceived higher quality of the private health sub system, the choice of provider and waiting times, which one can argue are behind the decision of users for choosing ISAPRE.

Legislation in Germany precludes substantial rises in premiums by limiting increases in premiums to occasions where they are necessary to maintain the financial viability of the insurer. For instance, since 2001 insurers must charge new subscribers to build up sufficient ageing reserves. In Chile, private health insurance premiums are certainly higher than contributions to FONASA, usually by a significant margin. This problem is compounded by the substantial amount of co-payments privately insured patients are required to make (although co-payments also apply under FONASA, particularly in the ‘free choice’ tiers). Fischer et al. [68] demonstrated that, in 2005, co-payments accounted for about one third of total expenditure for health for privately insured individuals, compared to about 15% for patients using FONASA. However, financial protection has increased substantially in recent years. In the absence of any regulation of the benefits package, the range of services covered varied widely and many patients were left with significant gaps in insurance coverage.

Another characteristic of Germany and Chile’s insurance market is that the mix of public and private providers varies, as does the relationship between providers and payers. In Germany, both statutory health insurance and private health insurance largely provide access to the same type of providers, regardless of their ownership status. FONASA members in Chile, in contrast, mainly receive services from public providers, unless they are prepared to pay extra for additional choice in a pay as you go voucher scheme. Privately insured patients usually seek care from private providers, although they can receive care in the public sector at any time if they lose private coverage.

Critics of Germany’s dual insurance system argue that substitutive private health insurance undermines equity in the health system. Similar criticism has been voiced repeatedly in Chile, as privately insured patients typically have significantly faster access to a wider range of services [69, 70]. Privately insured patients in Chile also typically pay a substantially higher price than they would for statutory health insurance. However, individuals with private insurance enjoy more choice and better access to health services, which does not correspond with the principle of ‘equal access for equal need’, even if it does involve higher costs. While various studies on waiting times in Germany differ in their conclusions, the variation in access to care in Germany is moderate compared with that of Chile. The variation in access within Chile has repeatedly been named as a cause of major inequalities in health care utilisation and health outcomes [69–71]. In the 2006 Chile’s Household Survey (Encuesta de Caracterización Socioeconómica Nacional - CASEN), the utilisation of health services was 30% higher in the wealthiest than in the poorest quintile of the population [72]. A 2003 study also found that those with the lowest income had the worst self-rated health [73].

Risk segmentation between statutory health insurance and the private insurance market and its relation to equity has been a major concern in both Germany and Chile. In both countries, the possibility for high-income earners to choose between statutory health insurance and private health insurance has led to substantial segmentation of risks between the two types of insurance. In Germany and Chile, the regulatory framework further exacerbates this tendency, as private health insurers are allowed (apart from applicants eligible for the basic tariff) to reject applications for cover and risk rate premiums, exclude cover of pre-existing conditions, charge extra for dependants and offer discounted premiums in exchange for high deductibles. This issue was partially addressed in Chile through recent reforms, such as the introduction of a risk equalisation scheme between private insurers, the regulation of price changes and the introduction of ‘explicit guarantees’. However, the evidence of the effect of these changes is still lacking and some of the measures, such as a risk equalization scheme, have not yet been consistently implemented. The consequence of this is that the substitutive market in both countries enjoys a high concentration of ‘low risks’, while the public sector covers a disproportionate number of ‘high risks’ – notably women and children, older people and individuals with larger families. In Germany, in 2013, about 51% of the privately insured were men, while women and children accounted for 31% and 18%, respectively [38]. In the case of Chile, the participation of the oldest and poorest quintile of the population in the ISAPRE system is below 5% [27]. Based on data from the 2000 and the 2006 CASEN household surveys [74], the probability of taking out private insurance increases with a higher level of income and decreases with higher risk of ill-health. Those who had taken out private insurance were also likely to live in urban areas and to be better educated than those reliant on statutory health insurance [72].

The Netherlands is a case in which a country with co-occurring public and private health insurance has sought to address some of the problems raised here. In 2006, the Netherlands underwent a market-oriented reform of its health care system through the Healthcare Market Structuring Act *(*Zorgverzekeringswet) which eliminated the division between public, statutory health insurance and private health insurance, yielding a universal basic tariff with comprehensive coverage of health services provided solely by competing private insurers [75]. Some immediate effects on the health insurance market were noted, such as a relatively high rate of consumer mobility among benefit designs in 2006 with some 18% switching plans and consolidation among health insurers from 59 to 26 between 2005 and 2014 [75]. Nevertheless, comparisons between the Dutch case and Germany or Chile are difficult; as we have noted, the political barriers to reform are high in both Germany and Chile and, consequently, reform on the scale of the Dutch case is unlikely at present.

Despite prima facie similarities between the German and Chilean arrangements between private health insurance and statutory social health insurance, unique developments in each country respectively have contributed to divergent approaches to addressing issues such as financing and regulation as we have discussed. Consequently, direct comparative analysis between these two systems is necessarily contingent on the acknowledgement of differences between Germany and Chile, not only involving the health care system but also intangible factors such as patient preferences, consumer expectations, and client orientation of health services in each country. Nevertheless, in this policy analysis, we have attempted to demarcate specific indicators of performance in the health sector, namely, horizontal equity, progressivity, and productive efficiency, to provide our analysis with more generalised, comparable measures. A more direct study of clinical populations in Germany and Chile, for instance, members of comparable health plans, could provide greater force to the arguments presented here through the generation of empirical data; however, given the relative complexity of national or supranational regulations on patient data protection and the challenges of reconciling international, multicentre studies with these regulations, we have sought to balance a more general policy analysis with the inclusion of empirical data from other studies.

Overall giving consumers a choice of public or private health insurance, as is the case in Germany and in Chile, is similar in effect to offering a choice of more than one plan. It is likely to result in two main outcomes: a market segmented by degree of risk, and financial barriers to private coverage for high risks. Negative effects can be diminished, to some extent by careful policy design, or can be addressed by regulation. Examples abound and range from abolishing choice and making social health insurance compulsory for the whole population, to introducing more incremental measures to tackle risk selection and increase access to coverage, such as cross-subsidies from private to public insurance, or tighter regulation of insurers, including risk adjustment. In both countries, the issue of risk selection remains unresolved. The loss of ‘high income’ users to the GKV continues to undermine the idea of equity and solidarity on which the German social security system rests. There have also been long-standing concerns about two-class medicine (Zweiklassenmedizin) and large parts of the population are uncomfortable with the idea that people with higher incomes should receive better care. In the past, efforts to abolish substitutive private health insurance were consistently opposed by an alliance of the medical profession, the Free Democrats (Freie Demokratische Partei - FDP) and large parts of the Christian Democratic Party (Unionsparteien - CDU/CSU). However, increasing unhappiness among the privately insured may help trigger reform in the years to come [76].

## Conclusions

The choice of private insurance in Germany and Chile has had far-reaching consequences on equity and efficiency in their respective health systems. Most of these consequences are due to serious market failures secondary to asymmetries of information that tend to negatively affect the efficiency and equity of these countries health systems. Although the comparative analysis presented was based on the broad definitions of equity and efficiency used by Thomson et al. [4, 7] and the OECD framework of insurance analysis [5, 19], the observed policy outcomes of a dual health insurance system for both Germany and Chile is a decreased horizontal and vertical equity of access and finance. When selective private health insurance was compared against universal public statutory coverage mandatory for all in our analysis, we observed that users were treated unequally based on how their contributions to state insurance or private premiums were determined by socio-demographic features such as health status, income, age, household size. In both countries having a dual insurance system has decreased the degree of progressivity in financing health care by allowing wealthier users to be pooled separately and hence pay proportionately less than those with lower incomes. In both systems, productive efficiency has also been seriously compromised given that competition has been historically based on risk selection rather than price or quality. The comparative extent and level of segmentation of both countries is not clear, but we can assume that in Chile segmentation will be more important than in Germany as a direct consequence of higher levels of inequity existing in the country. Therefore, a comparative quantitative analysis as to the extent of segmentation levels in both countries is crucial as to understand the phenomenon exposed here. Risk segmentation has also had serious consequences for productive efficiency due to the tendency of private insurers in both Germany and Chile to not reduce operating costs by lowering their administrative costs, but instead vertically integrating with providers and suppliers. Some attempts have been made in Chile to tackle the impact of substitutive private insurance on equity and efficiency, but the long-term consequences of these reforms have yet to be fully established. There has been less pressure to reform the dual insurance system in Germany in recent years, as economic growth and lower unemployment have meant financial surpluses for the GKV. However, the long-term challenges to the sustainability and acceptability of a system that privileges the better-off at the expense of the efficiency of the system are likely to remain on the agenda of policy-makers in the years to come. This is specially so when introducing elements of private coverage into public systems in health systems such as the British National Health Service, single payers, or Social Health Insurance.

## Additional file

Additional file 1: PRISMA Flow Diagram. Private Health Insurance in Germany and Chile: Two Stories of Co-Existence, Segmentation and Conflict. (DOC 48 kb)

## Abbreviations

CAEC: Cobertura Adicional Enfermedades Catastrophicas (Aditional Coverage for Catastrophic Events)
CASEN: Encuesta de Caracterización Socioeconómica Nacional (national Socioeconomic Characterisation Survey)
CBS: Conjunto De Beneficios Sociales (Cluster Of Health Benefits)
CDU: Christlich Demokratische Union Deutschlands (Christian Democratic Union of Germany)
CSU: Christlich-Soziale Union in Bayern (Christian Social Union in Bavaria)
DALY: Disability Adjusted Life Years
DRG: Diagnostic Related Grouping
FDP: Freie Demokratische Partei (Free Democratic Party)
FONASA: Fondo Nacional de Salud (Social Health Insurance)
GDP: Gross Domestic Product
GES: Garantias Explicitas en Salud (Explicit Health Guarantees)
GKV: Gesetzliche Krankenversicherung (Social Health Insurance)
HMO: Health Management Organisation
ISAPRE: Instituto de Salud Previsional (Private Health Insurance)
MINSAL: Ministerio de Salud (Chilean Health Ministry)
MLE: Modalidad Libre Eleccion (Free Choice Modality)
OECD: Organisation for Economic Co-operation and Development
OOP: Out of Pocket Payments
PKV: Privaten Krankenversicherung (Private Health Insurance)
SGB V: Sozialgesetzbuch (Social Code Book V)
WHO: World Health Organization
