# Supplementary material for: Private health insurance in Germany and Chile: two stories of co-existence, segmentation and conflict
Source: Int J Equity Health. 2018 Aug 3;17:112. doi: 10.1186/s12939-018-0831-z (PMC6091104; doi:10.1186/s12939-018-0831-z)
Supplement: Supplementary file 1 — PRISMA Flow Diagram. Private Health Insurance in Germany and Chile: Two Stories of Co-Existence, Segmentation and Conflict. (DOC 48 kb) [file 12939_2018_831_MOESM1_ESM.doc]

**PRISMA Flow Diagram**

**Private Health Insurance in Germany and Chile: Two Stories of Co-Existence, Segmentation and Conflict**

**Screening**

**Included**

**Eligibility**

**Identification**

Records identified through database searching (PubMed & Google Scholar)
(n = 24)

Additional records identified through other government sources
(n = 55)

Records after duplicates removed
(n = 79)

Records screened
(n = 79)

Records excluded
(n = 4)

Full-text articles assessed for eligibility
(n = 75)

Full-text articles excluded, with reasons
(n = 3)

Studies included in qualitative synthesis
(n = 72)

Studies included in quantitative synthesis
(No quantitative analysis was performed)

Studies and documents included:

1. Frenz P, Delgado I, Kaufman JS, Harper S. Achieving effective universal health coverage with equity: evidence from Chile. Heal Policy Plan . 2014;29:717–31.

2. Busse R, Riesberg A. Health Systems in Transition: Germany. Copenhagen; 2004.

3. Dawes Ibáñez A, Gallego F. Health care reform and its effect on the choice between public and private health insurance: evidence from Chile. Pontif Univ Catol Chile, Inst Econ Santiago. 2010.

4. Thomson S, Busse R, Crivelli L, van de Ven W, Van de Voorde C. Statutory health insurance competition in Europe: a four-country comparison. Health Policy (New York). 2013;109:209–25.

5. Böhm K, Schmid A, Götze R, Landwehr C, Rothgang H. Five types of OECD healthcare systems: Empirical results of a deductive classification. Health Policy (New York). 2013;113:258–69.

6. Rothgang H, Cacace M, Grimmeisen S, Wendt C. The changing role of the state in healthcare systems. Eur Rev. 2005;13 SUPPL. 1:187–212.

7. Thomson S, Mossialos E. Choice of public or private health insurance: learning from the experience of Germany and the Netherlands. J Eur Soc Policy . 2006;16:315–27.

8. Sapelli C. Risk segmentation and equity in the Chilean mandatory health insurance system. Soc Sci Med. 2004;58:259–65.

9. Pedraza CC, Toledo LP. El gasto de bolsillo en salud: el caso de Chile, 1997 y 2007. Pan Am J Public Heal. 2012;31.

10. Wendt C, Frisina L, Rothgang H. Healthcare System Types: A Conceptual Framework for Comparison. Soc Policy Adm. 2009;43:70–90.

11. Anckar C. On the Applicability of the Most Similar Systems Design and the Most Different Systems Design in Comparative Research. Int J Soc Res Methodol. 2008;11:389–401 doi:10.1080/13645570701401552.

12. Atella, V. and Spandonaro F. Private Health Insurance in Italy: Where We Stand Now. Euro Observer. 2004;:6(1): 6-7.

13. World Bank. Voluntary (Private) Opt-out Insurance: Is it Good for Russia? Washington, DC.

14. Propper C, Rees H, Green K. The Demand for Private Medical Insurance in the UK: A Cohort Analysis. Econ J. 2001;111:180–200.

15. Mooney GH. Equity in health care: confronting the confusion. Eff Health Care. 1983;1:179–85.

16. Culyer AJ, Wagstaff A. Equity and equality in health and health care. J Health Econ. 1993;12:431–57.

17. Wagstaff A, van Doorslaer E. Income Inequality and Health: What Does the Literature Tell Us? Annu Rev Public Health. 2000;21:543–67.

18. Organization WH. The world health report 2000: health systems: improving performance. World Health Organization; 2000.

19. OECD. Proposal for a taxonomy of health insurance. Paris; 2004.

20. Barr N. The Welfare State as Piggy Bank: Information, Risk, Uncertainty, and the Role of the State: Information, Risk, Uncertainty, and the Role of the State. OUP Oxford; 2001.

21. Barr NA. The economics of the welfare state. Stanford University Press; 1998.

22. Barr N. Economic theory and the welfare state: a survey and interpretation. J Econ Lit. 1992;30:741–803.

23. Huber E, Pribble J. Social Policy and Redistribution under Left Governments in Chile and Uruguay. 2011.

24. Unger J-P, De Paepe P, Cantuarias GS, Herrera OA. Chile’s Neoliberal Health Reform: An Assessment and a Critique. PLoS Med. 2008;5:e79.

25. Raczynski D. Social Policies in Chile: Origin, Transformations, and Perspectives. Democr Soc Policy Ser Univ Notre Dame Indiana. 1994.

26. Miranda E. Evolución y Perspectivas: La Salud en Chile. Miranda E (ed). 1994. La Salud en Chile: Evolución y Perspectivas . Santiago: Centro de Estudios Públicos.; 1994.

27. Barrientos A, Lloyd-Sherlock P. Reforming health insurance in Argentina and Chile. Health Policy Plan. 2000;15:417–23.

28. Missoni E, Solimano G. Towards universal health coverage: the Chilean experience. World Health Report, Background Paper 4.; 2010.

29. Bitran R. Explicit health guarantees for Chileans: the AUGE benefits package. 2013.

30. Letelier LM, Bedregal P. Health reform in Chile. Lancet (London, England). 2006;368:2197–8.

31. Tribunal de Defensa de la Libre Competencia. La Corte Suprema confirmó la sentencia del TDLC que absolvió a cinco Isapres del cargo de colusión. Tribunal de Defensa de la Libre Competencia. 2008.

32. Ärztezeitung. Krankenversicherung : Beiträge sind ab 2010 komplett absetzbar. Ärztezeitung. 2009.

33. Agostini C, Saavedra E, Willington M. Collusion in the Private Health Insurance Market: Empirical Evidence for Chile. Ilades-georget Univ Work Pap. 2008.

34. Palmucci GA, Dague L. The Welfare Effects of Banning Risk-Rated Pricing in Health Insurance Markets: Evidence from Chile. 2015.

35. OECD. OECD Health Data 2015. 2015.

36. OECD. Income Stadistics - OECD Data. OECD. 2014.

37. Statistisches Bundeamt. Sozialleistungen. Angaben zur Krankenversicherung (Ergebnisse des Mikrozensus). Wiesbaden.

38. PKV. Zahlenbericht der Privaten Krankenversicherung 2013. Cologne; 2014.

39. BMG. Daten des Gesundheitswesens 2013. Bundesministerium für Gesundheit. 2013.

40. PKV. Zahlenbericht der Privaten Krankenversicherung. Cologne; 2017; 2016.

41. BMG. Entkopplung der Gesundheitskosten vom Arbeitsmarkt. Bundesministerium für Gesundheit. 2011.

42. Comisión Asesora Presidencial. Estudio y Propuesta de un Nuevo Marco Jurídico para el Sistema Privado de Salud. Santiago, Chile.; 2014.

43. Superintendencia de Salud. Estadísticas consolidada de cartera del sistema Isapre año 2015. Biblioteca Digital, Superintendencia de Salud. Chile. 2015.

44. Sánchez M. Análisis de los Planes de Salud del Sistema Isapre. Santiago; 2014.

45. Departamento de Estudios y Desarrollo. El Mercado de los Seguros Complementarios de Salud. Santiago, Chile.; 2008.

46. OECD. Out-of-pocket expenditure on health, Health: Key Tables from OECD, No. 5.

47. OECD. Health at a Glance 2015. In: OECD Indicators. Paris: OECD Publishing; 2015.

48. Cid C, Muñoz A, Riesco X, Inostroza M. Equidad en el financiamiento de la salud y protección financiera en Chile: una descripción general. Cuad Médicos Soc. 2006;46:5–12.

49. Castillo-Laborde C, Villalobos Dintrans P. Caracterización del gasto de bolsillo en salud en Chile: una mirada a dos sistemas de protección. Rev Médica Chile; Vol 141, núm 11 NOVIEMBRE 2013. 2013.

50. PKV. Der private Krankenversicherungsschutz im Sozialrecht. Cologne; 2015.

51. DKV. Versicherungslexikon der Deutschen Krankenversicherung. 2008.

52. Superintendencia de Pensiones. Aumento de topes imponibles para el cálculo de cotizaciones 2015 - Prensa. Superintendencia de Pensiones. Superintendencia de Pensiones Informa. Chile. 2015.

53. Bastías G, Pantoja T, Leisewitz T, Zárate V. Health care reform in Chile. Can Med Assoc J. 2008;179:1289–92.

54. Banmedica. Un Plan para Ti: Simulador y busca un plan. 2015.

55. Mercurio. Planes de Isapre suben de precio, bajan las coberturas y aumentan las restricciones. Economia y Negocios. 2014.

56. Superintendencia de Salud. Estadísticas Financieras de las Isapres a marzo de 2015. Biblioteca Digital, Superintendencia de Salud. Chile. 2015.

57. PKV. Gesundheitsreform 2007, Neuregelungen für die PKV. Verband der Privaten Krankenversicherung. 2008.

58. Waldendzik A, Greβ S, Manouguian M, Wasem J. Vergütungsunterschiede im ärztlichen Bereich zwischen PKV und GKV auf Basis des standardisierten Leistungsniveaus der GKV und Modelle der Vergütungsangleichung. 2008.

59. Murray SF. Relation between private health insurance and high rates of caesarean section in Chile: qualitative and quantitative study. BMJ. 2000;321:1501–5.

60. OECD. Health at a Glance 2017. 2017.

61. Superintendencia de Salud. Prestadores de Salud, Isapres y Holdings: ¿Relación Estrecha? 2013.

62. Valencia R. Esta es la concentración de las Isapres que explica altas utilidades. La Nacion. 2012.

63. Busse R, Blümel M, Knieps F, Bärnighausen T. Statutory health insurance in Germany: a health system shaped by 135 years of solidarity, self-governance, and competition. Lancet. 2017;390:882–97.

64. Castillo Taucher C. Presentación / Modifica el Sistema Privado de Salud, incorporando un plan garantizado. (Ministerio de Salud). Comision de Salud, Senado de Chile. 2015.

65. Guichou GS. ¿Tienen resultados las comisiones formadas por Michele Bachelet? La Nacion. 2015.

66. Aguilera C. Las Comisiones Asesoras Presidenciales del Gobierno de Michelle Bachelet. Santiago de Chile; 2009.

67. Maarse H, Bartholomée Y. A public--private analysis of the new Dutch health insurance system. Eur J Heal Econ. 2007;8:77–82.

68. Fischer R, González P, Serra P. Does competition in privatized social services work? The Chilean Experience. World Dev. 2006;34:647–64.

69. Holst J, Laaser U, Hohmann J. Chilean health insurance system: a source of inequity and selective social insecurity. J Public Health (Bangkok). 2004;12.

70. Zuckerman E, Kadt E De. The Public-Private Mix in Social Services: Health Care and Education in Chile, Costa Rica, and Venezuela. Interam Dev Bank Washingt DC. 1997.

71. Lüngen M, Stollenwerk B, Messner P, et al. Waiting times for elective treatment according to insurance status: A randomized empirical study in Germany. Int J Equity Health. 2008;7.

72. Encuesta Casen. No Title. Observatorio Social - Ministerio de Desarrollo Social, Gobierno de Chile. 2006.

73. Subramanian S V, Delgado I, Jadue L, Vega J, Kawachi I. Income inequality and health: multilevel analysis of Chilean Communities. J Epidemiol Community Health. 2003;57:844–848.

74. Dawes I. Health Care Reform and its effect on the Choice between Public and Private Health Insurance: Evidence from Chile. Pontificia Universidad Catolica de Chile, Instituto de Economia; 2010.

75. Ministerio de Desarrollo Social. Encuesta CASEN 2006-Portal de Datos Públicos - Dataset. Portal de Datos Públicos - Dataset. Chile. 2013.

76. Maarse H, Jeurissen P, Ruwaard D. Results of the market-oriented reform in the Netherlands: a review. Heal Econ Policy Law. 2016;11:161–178.

77. Zeit Online. Nie mehr zweite Klasse. Zeit Online. 2012.
